# Evaluation of the feasibility of cardiac gating for SBRT of ventricular tachycardia based on real‐time ECG signal acquisition

**DOI:** 10.1002/acm2.13814

**Published:** 2022-10-26

**Authors:** Cristiano Q. M. Reis, James L. Robar

**Affiliations:** ^1^ Department of Radiation Oncology Dalhousie University Halifax Nova Scotia Canada; ^2^ Department of Medical Physics Nova Scotia Health Halifax Nova Scotia Canada; ^3^ Department of Physics and Atmospheric Science Dalhousie University Halifax Nova Scotia Canada

**Keywords:** real‐time cardiac gating, SBRT, ventricular tachycardia

## Abstract

**Purpose:**

To investigate the feasibility of cardiac synchronized gating in stereotactic body radiation therapy (SBRT) of ventricular tachycardia (VT) using a real‐time electrocardiogram (ECG) signal acquisition.

**Methods and materials:**

Stability of beam characteristics during simulated ECG gating was examined by developing a microcontroller interface to a Varian Clinac iX linear accelerator allowing gating at frequencies and duty cycles relevant to cardiac rhythm. Delivery accuracy was evaluated by measuring dose linearity with an ionization chamber, and flatness and symmetry with a two‐dimensional detector array, for different gating windows within typical human cardiac cycle periods. To establish a practical method of gating based on actual ECG signals, an AD8232 Heart Monitor board was used to acquire the ECG signal and synchronize the beam delivery. Real‐time cardiac gated delivery measurements were performed for a single 10 × 10 cm^2^ field and for a VT‐SBRT plan using intensity‐modulated radiation therapy (IMRT).

**Results and discussion:**

Dose per monitor unit (MU) values were found to be linear within most gating windows investigated with maximum differences relative to non‐gated delivery of <2% for gating windows ≥200 ms and for >10 MUs. Beam profiles for both gated and non‐gated modes were also found to agree with maximum differences of 0.5% relative to central axis dose for all sets of beam‐on/beam‐off combinations. Comparison of dose distributions for intensity‐modulated SBRT plans between non‐gating and cardiac gating modes provided a gamma passing rate of 97.2% for a 2% 2 mm tolerance.

**Conclusions:**

Beam output is stable with respect to linearity, flatness, and symmetry for gating windows within cardiac cycle periods. Agreement between dose distributions for VT‐SBRT using IMRT in non‐gated and cardiac cycle gated delivery modes shows that the proposed methodology is feasible. Technically, gating for delivery of SBRT for VT is possible with regard to beam stability.

## 1 INTRODUCTION

Ventricular tachycardia (VT) is a cardiac arrhythmia that can lead to sudden death.^1^ Conventional treatment techniques, including the use of anti‐arrhythmic drugs,^2^ implantable cardioverter defibrillator,^3^ and catheter ablation with radiofrequency,^4^ are associated with a high rate of recurrence of VT episodes.^5^, ^6^, ^7^ Stereotactic body radiation therapy (SBRT) with one single fraction of 25 Gy prescribed to the arrhythmogenic scar region of the heart has become an attractive noninvasive approach for treating VT.^8^, ^9^, ^10^, ^11^, ^12^, ^13^ Recent results published in the literature have shown promising outcomes demonstrating that patients have complete or near complete resolution of VT after treatment with radiation.^10^, ^12^, ^14^ However, as a novel technique, VT‐SBRT still lacks standardized procedures,^15^ for example, for target volume delineation, treatment planning, and management of respiratory and cardiac motion.

VT targets and nearby organs at risk (OARs) are not static due to respiratory and cardiac motions, which increases their position uncertainty. Studies have shown that intrinsic cardiac motion has a significant impact on the total heart displacement (respiration plus cardiac motion), with some regions of the heart presenting mean motion amplitude of up to 17 mm (13–17 mm) within the period of one cardiac cycle.^16^, ^17^ Therefore, it is important to understand and incorporate strategies to manage cardiac motion for treating VT using SBRT.^18^

A straightforward approach for managing the motion issue in VT‐SBRT would be to encompass the entire range of the target motion within the volume to be treated, that is, to form an internal target volume (ITV) similar to that performed for lung SBRT.^19^ However, by design this ITV approach knowingly increases the irradiated volume and consequently the exposure of healthy tissues. Another common approach to handle respiratory motion in lung SBRT is a gating technique where the target volume is irradiated only when it enters a predefined phase or amplitude within the respiratory cycle. Commonly, the timing of respiratory gating is based on an external respiration surrogate or internal fiducial markers.^19^

Analogous to respiratory gating, the purpose of this work was to develop a methodology that would allow one to gate the radiation beam during SBRT of VT based on a real‐time acquisition of the patient's electrocardiogram (ECG) signal, and to synchronize beam delivery with a specific phase of the cardiac cycle. Using this methodology, our goal was to irradiate the heart at the quiescent intervals of the cardiac cycle where the motion of the heart is minimal, that is, during ventricular diastole.^20^ In terms of frequency and duty cycle (d.c.), this operation does not correspond to common ranges normally used in the clinic. Therefore, we begin this study by evaluating beam output stability with regard to dose linearity, beam flatness, and symmetry, for several gating windows within typical cardiac cycle periods. We then demonstrate a synchronized gating methodology with an acquisition of actual ECG signals. Using this approach, we deliver example intensity‐modulated radiation therapy (IMRT) treatments for SBRT of VT with and without cardiac gating to assess dose distribution accuracy.

An approach for gating the beam delivery for SBRT of VT has recently been published by Poon et al.^21^ In their study, they proposed an indirect method of controlling the time of beam‐on and beam‐off by using the MLC motion to control timing for a volumetric modulated arc therapy (VMAT) delivery. Beam delivery synchronization is maintained by adjusting LINAC parameters according to heart rate variations. However, as pointed out in their manuscript, they were not able to perform a real‐time synchronization based on live ECG acquisition as the clinical system demands calculations of all plan parameters before delivering the treatment. In our present work, the beam is gated via a logical signal sent directly to the LINAC with synchronization to the cardiac cycle based on real‐time acquisition of ECG signal.

## 2 MATERIALS AND METHODS

### 2.1 Methodology of beam gating at cardiac frequencies

A set of gating windows was defined to evaluate beam output characteristics under high‐frequency‐gated delivery mode in comparison to non‐gated delivery. A 6 MV beam from a Clinac iX linear accelerator (Varian Medical Systems, Inc., Palo Alto, USA) was gated using an Elegoo UNO R3 Arduino microcontroller board (Elegoo, Shenzhen, Guangdong, CN) to provide a logical signal (0 or 5 V) to the LINAC that could depolarize the gridded electron gun (usually at a frequency of roughly 700 MHz)^22^, ^23^ and hold off the electron current. This allowed beam gating at frequencies typical of heart rates between 60 and 120 (or greater) beats/min. It is important to note that although patients undergoing VT episodes can experience much higher heart rates (170 bpm or more), VT‐SBRT is generally not performed during a VT episode. Beam‐on and beam‐off times were defined for the cardiac cycle periods (*τ*) as shown in Table 1. Beam‐on times between 200 and 400 ms were used to analyze the beam output for cardiac cycle periods between 500 and 750 ms. For a cardiac cycle period of 1000 ms, beam‐on times ≥400 ms were used to avoid using very low d.c. Additionally, as it is explained in the next sections, the quiescent phase of the heart, during which the target should be irradiated, lasts approximately one third of the cardiac cycle. Therefore, although interesting for testing the limits of the LINAC, short beam‐on times (e.g., d.c. ≤20%) are unlikely to be used when gating VT‐SBRT.

**TABLE 1:** Beam‐on and beam‐off times used to gate the 6 MV beam for different cardiac cycle periods

Cardiac cycle period (heart rate)	τ = 500 ms (120 beats/min)	τ = 600 ms (100 beats/min)	τ = 750 ms (80 beats/min)	τ = 1000 ms (60 beats/min)
**Gating window (beam‐on, beam‐off)**	(200, 300 ms)	(200, 400 ms)	(200, 550 ms)	(400, 600 ms)
	(300, 200 ms)	(300, 300 ms)	(300, 450 ms)	(500, 500 ms)
	(400, 100 ms)	(400, 200 ms)	(400, 350 ms)	(600, 400 ms)

### 2.2 Beam output measurements

#### 2.2.1 Dose chamber linearity

To investigate the dose linearity of the 6 MV photon beam output under cardiac gating conditions, a PTW 31010 mini chamber (PTW, Freiburg, Germany) with 0.125 cm^3^ volume and a SuperMAX Electrometer (Standard Imaging, Middleton, WI, USA) were used. Measurements were performed for monitor units (MUs) ranging from 10 to 200 MU, with the dose rate set to 600 MU/min, and with the ionization chamber located at 5 cm depth in solid water with SSD = 95 cm and a field size of 10 × 10 cm^2^. MU settings of 10, 20, 30, 40, 50 100, 150, and 200 MU were used, and the chamber response per MU was obtained by dividing ionization readings with the MUs delivered. Each measurement was repeated three times to provide an average output per MU, for the non‐gated mode and for the range of gating windows given in Table 1. The Task Group 142 report^24^ from the American Association of Physicists in Medicine recommends both beam energy and output constancy as important tests for gated accelerator operation. For simplicity, beam output constancy was chosen to be evaluated in this work, instead of beam energy constancy that would demand acquiring double chamber reading at two distinct depths.

#### 2.2.2 Dose profile evaluation

Comparison of beam output between non‐gated and gated deliveries was performed by measuring beam dose profiles of the 6 MV photon beam. For this purpose, an IBA I'mRT MatriXX detector array, located within a MultiCube phantom (IBA Dosimetry Schwarzenbruck, Germany), was used for measuring two‐dimensional dose distributions. The OmniPro‐I'mRT software version 1.7 (IBA Dosimetry Schwarzenbruck, Germany) was used to evaluate dose profiles and assess beam flatness and symmetry.

Commonly, beam flatness is measured in a water phantom at 10 cm depth and an SSD of 100 cm with the largest field size available, whereas symmetry is usually measured at a depth of maximum dose (*d*_max_) and for the largest field size. In this work, all dose profiles’ measurements were performed with the matrix detectors inserted into the MultiCube phantom aligned to the machine isocenter with SSD = 90 cm and a field size of 10 × 10 cm^2^. This locates the detector array at 10 cm depth within the phantom. For practical reasons, a single depth of 10 cm was chosen for performing all measurements. Additionally, a 10 × 10 cm^2^ field was used as we are interested in treatments fields that will be <10 cm in dimension. Measurements were performed for non‐gated and gated beam delivery according to beam‐on and beam‐off times shown in Table 1 and using 100 MUs. For analysis, all dose profiles were normalized to the dose at the central axis.

### 2.3 Beam gating synchronization with ECG signal

In this step, we developed a methodology that allowed us to gate the beam delivery in synchronization with the cardiac cycle based on a real‐time acquisition of an ECG signal. Using the method described herein, the beam may be gated on and off at specific phases of the cardiac cycle, and the duration of irradiation can be adjusted dynamically, based on variations of the heart rate. For acquiring a live ECG signal, a Single Lead AD8232 Heart Rate Monitor ECG Development Kit (Analog Devices, Wilmington, MA, USA) was used and interfaced with an Arduino microcontroller board, which in turn provided logical signals to the gating input of the linear accelerator. Latency between the ECG signal acquisition and sending a beam on/off signal by the Arduino board was assessed using a TDS 2014C (Tektronix, Beaverton, USA) four channels digital storage oscilloscope and found to be ∼1 ms.

A crucial step for determining the cardiac cycle is the identification of the QRS complex. The cardiac cycle starts with the depolarization (contraction) of the atria stimulated by the sinoatrial node and represented by the P wave in the ECG signal. Subsequently, the QRS complex visualized in the ECG represents the depolarization of the ventricles at the beginning of systole, or ventricular contraction. The following stage is marked by the ventricular repolarization, identified in the ECG by the T wave, and marking the end of systole and beginning of diastole (relaxation) of the ventricles. According to the extensive research by Pan and Tompkins,^25^ QRS complex identification is quite challenging due to its physiological variability in the presence of noise from muscles, electrode motion artifacts, electromagnetic interference, and high frequency T waves. A methodology to account for heart rate variability and determine the cardiac cycle period was employed as described later.

#### 2.3.1 Previous ECG signal acquisition and processing

Before gating the beam, an ECG signal was acquired for 30 s from a normal volunteer using the AD8232 Heart Rate Monitor ECG and the Elegoo UNO R3 boards connected to a computer. Three disposable electrodes located on the chest were connected to the AD8232 Heart Monitor for acquiring ECG data. Electrodes were positioned as follows: The red electrode was located under right clavicle within the rib cage near the right arm; the yellow electrode was placed under left clavicle within the rib cage near the left arm; and green electrode was positioned on the right side on the lower edge of the rib cage close to right leg.

**FIGURE 1:**
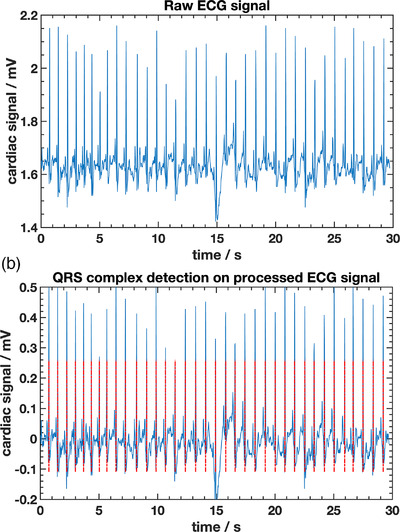
Electrocardiogram (ECG) signal acquired using the AD8232 heart monitor. In (a), a raw signal is shown from one acquisition of 30 s duration, which is then processed using the Pan–Tompkins algorithm to correctly identify the QRS complex as shown in (b) with dashed lines overlapping the processed signal.

An algorithm was implemented using MATLAB (R2019a, MathWorks, Inc.) to read, visualize, and store the ECG data. Figure 1a shows the raw ECG signals acquired for 30 s using the AD8232 heart monitor and the MATLAB subroutine. The cardiac cycle is defined as the time between two consecutives R‐peaks. An open source code^26^ in MATLAB was used to process the signal and correctly identify the R‐peaks as shown in Figure 1b. This code is an implementation of the Pan–Tompkins algorithm^25^ that is one of the most widely used algorithms and most frequently cited approaches for the extraction of QRS complexes from an ECG signal.^27^, ^28^ The MATLAB subroutine used to identify the R‐peaks was tested using 10 different known ECG signals (including signals with VT episodes) randomly selected from the widely used MIT‐BIH Arrhythmia Database.^29^ The subroutine was able to correctly identify the R‐peaks of the recorded ECG signals, and values of cardiac cycles and heart rates calculated based on R–R intervals were within the expected range provided in the database. Variation of the cardiac cycle period with significant decreasing during VT episodes was also correctly identified by the subroutine.

The cardiac cycle period can be determined as an average of the best R–R intervals in the ECG signal after stabilization is reached.^30^ Under normal circumstances and in steady rhythm, the cardiac cycle should be approximately constant without presenting large variations among different R–R intervals. In this work, the best R–R intervals were defined as a set of the 10 last arbitrarily chosen R–R intervals with a similar period. An average of the last 10 R–R intervals was used to determine a mean cardiac cycle value $\bar{\tau }$ and use it as a reference value to evaluate if the real‐time cardiac cycle, assessed during beam gating, will be within an acceptable range of variation.

The rationale for previously identifying the QRS complex is to help avoiding false R‐peak detection when gating the beam based on the ECG signal amplitude, while also verifying if patient heartbeat is stable. Although the amplitude of the R‐peak is quite distinguishable from the rest of the ECG signal, an R‐peak identification, based solely on the amplitude, would not work properly due to disturbance caused, for example, by muscle contraction or electrode motion artifacts, that is, noise that can occur which would cause “false positives.” Any noisy high amplitude signal wrongly counted as an R‐peak would provide a very short cardiac cycle and would then be disregarded when compared to the previously determined mean cardiac cycle.

To estimate how much the heart rate can vary under normal conditions, a series of 20 ECG signals were acquired at different times of the day from the same normal volunteer. An average value of 731 ms was obtained with all individual measurements falling within ±15% variation around the average. In this sense, like the approach used by Leiner et al.,^30^ any value of τ measured within the interval $\bar{\tau }-15\%<\tau <\bar{\tau }+15\%$ would be considered accepted to account for heart rate variations during the beam gating. Any variation in the patient's heart rate outside of those intervals would be considered abnormal and the radiation beam should not be gated on until the values are within tolerance. In a clinical scenario, $\bar{\tau }$ can be determined for each patient in a similar way based on a training period with short ECG signal acquisitions of 30 s, although time to reach stability may be patient dependent.

#### 2.3.2 ECG‐synchronized gated beam delivery

**FIGURE 2:**
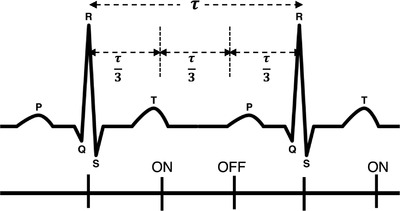
Illustration of the beam gating in synchronization with patient's heart rate. After the calculation of the cardiac cycle period τ (using the first two R‐peaks), the program waits for one third of the cardiac cycle after the last R‐peak detected (first QRS complex in the figure) to turn the beam on at approximately the end of the T wave. After one third of the cardiac cycle the beam is then turned off at the beginning of the P wave and will be turned on again at one third of the cardiac cycle after the next R‐peak (approximately two thirds of the cardiac cycle after being turned off).

To synchronize the beam delivery with a real‐time ECG signal, a methodology was developed to constantly evaluate the cardiac cycle and adjust the timing of beam‐on and beam‐off to account for heart rate variations. An application was developed using the Arduino Integrated Development Environment or Arduino Software for programing of the Arduino hardware microcontroller and communication with the AD8232 ECG board. The program begins by identifying the first two R‐peaks in the ECG signal and calculating the cardiac cycle period τ based on the time of the R–R interval. For values of τ within ±15% of the previous calculated reference value, $\bar{\tau }$, the beam is turned on at one third of a cardiac cycle (τ/3) after the last detected R‐peak (second one used to determine *τ*) and is held on for one third of the cardiac cycle. Following this, the beam is held off for two thirds of the cardiac cycle (2τ/3) as shown in Figure 2. The signal amplitude is again evaluated. If a new R‐peak is found, a new value of *τ* is calculated, based on this new R‐peak (third one) and the previous one (second one). If all conditions are met again, the irradiation cycle is repeated. This approach allows irradiation during the quiescent phase (ventricular diastole) of the heart as proposed by Poon et al.,^21^ where heart motion is minimal.^20^ Using values of τ within ±15% of $\bar{\tau }$, we also account for the 200 ms refractory period requirement as pointed out by Pan and Tompkins.^25^ As it is physiologically impossible for two consecutive R‐peaks to occur within a period of <200 ms, this refractory period helps to avoid false R‐peak detection. It is important to point out that VT patients may experience rapid heart rate changes, but it is not the goal of this methodology to treat the patient under such abnormal conditions. In that sense, any variation in the patient's heart rate outside of the limits defined before is considered abnormal and the radiation beam will not be turned on until the values are within tolerance.

After verifying correct operation of the application using the Arduino and AD8232 ECG board alone, the methodology was tested experimentally by creating and delivering a simple plan in Eclipse treatment planning system (TPS) (Eclipse v.15.6.06, Varian Medical Systems, Inc., Palo Alto, USA) consisting of a single 10 × 10 cm^2^ field with 15 Gy prescribed to the isocenter. This plan was delivered to the I'mRT MatriXX detector with the MultiCube phantom positioned isocentrically, that is, with the array at 10 cm depth, and using a real‐time ECG signal acquisition from a normal volunteer to gate the beam. The delivery accuracy of the real‐time ECG synchronized gating mode was evaluated by the comparison of the dose distributions with the same single field plan delivered in non‐gated mode. It is important to note that as VT radioablation is not expected to be performed with the patient undergoing a VT episode, we did not examine the case of an ECG signal containing cardiac arrhythmias. Furthermore, the focus of this work is to evaluate the beam characteristics and dose delivery in an ECG‐gated scenario considering the range of cardiac cycle periods that a patient can present.

#### 2.3.3 Real‐time cardiac–synchronized gated SBRT

As a main goal of this work was to show the feasibility of a real‐time cardiac synchronized gating for SBRT of VT, a realistic plan was created in Eclipse and delivered in gated and non‐gated modes. For planning, a thoracic CT data set with 0.5 mm × 0.7 mm × 0.7 mm voxel dimensions was sourced from a public repository^31^ and imported into Eclipse for contouring of the main OARs and an exemplary target volume. Following a previous approach,^32^ the target volume was delineated on the endocardium of the left ventricle. In this way, the contoured PTV would represent the final target volume to be treated similarly to an ITV technique as reported by Neuwirth et al.^12^ A seven‐field 6 MV sliding window IMRT plan was created in the Eclipse TPS with a single fraction dose of 25 Gy prescribed to the PTV and with the following field characteristics (gantry angle, MUs, number of segments): G0 (0°, 1468, 115), G51 (51°, 1413, 96), G102 (102°, 1440, 84), G153 (153°, 1525, 84), G204 (204°, 1985, 93), G255 (255°, 1638, 93), and G306 (306°, 1594, 98). The plan was optimized using the photon optimizer algorithm v.15.6.06 and dose was calculated using the Anisotropic Analytical Algorithm v.15.6.06 with a grid size of 0.2 cm. The SBRT plan was then delivered to the I'mRT MatriXX detector with the MultiCube phantom using the real‐time cardiac synchronized gating methodology as described before. Delivery accuracy was evaluated by comparison with non‐gated delivery mode.

An IMRT delivery technique was chosen for testing our proposed cardiac gating methodology in the Clinac iX because gated VMAT was first supported in Varian machines in the TrueBeam platform (Varian Medical Systems, Palo Alto, CA).^33^ Additionally, even using TrueBeam treatment units, currently there is no direct way of gating a VMAT or IMRT beam by sending a logical signal as we did in the Clinac iX without using a proprietary interface. An indirect method of controlling the time of beam on and beam off by using the MLC motion to control timing for the VMAT delivery has been demonstrated by Poon et al.^21^

## 3 RESULTS

### 3.1 MU linearity

**FIGURE 3:**
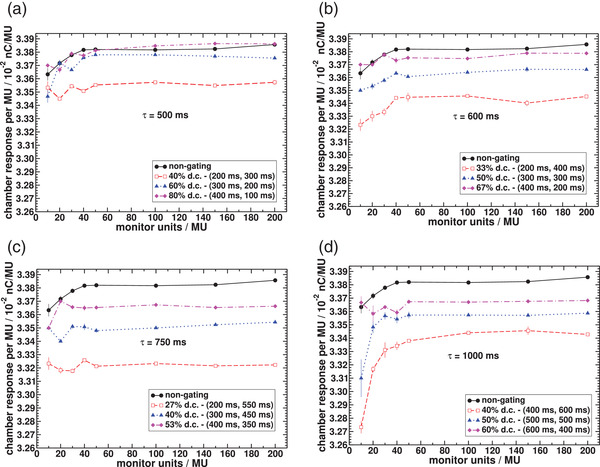
Chamber response per monitor unit (MU) for gated and non‐gated delivery and for cardiac cycle periods of 500 ms (a), 600 ms (b), 750 ms (c) and 1000 ms (d). A range of (beam‐on, beam‐off) times was evaluated for each cardiac cycle period. Error bars represent the standard deviation from the mean value in three repeated measurements.

Figure 3 compares the average response per MU (nC/MU) of the PTW ionization chamber between non‐gated beam delivery and several beam‐on and beam‐off time combinations for four different cardiac cycle periods. It was observed that for the non‐gated mode irradiation MU linearity was within 0.7% for MUs delivered between 10 and 200 MUs. For the gated mode linearity is within 1% from 10 to 200 MUs for all gating windows investigated except for the cardiac period of 1000 ms with d.c. of 40% and 50% where linearities are within 2% and 1.5% respectively.

Figure 3 also shows that chamber responses per MU with gating windows are relatively lower than the response obtained in non‐gated mode especially for low d.c. and for small amount of MUs. A maximum difference of 2.7% relative to non‐gated mode is found with (beam‐on, beam‐off) times of (400, 600 ms) and for 10 MUs as shown in Figure 3d. Differences compared to the non‐gated mode decrease with increasing the d.c. (beam‐on time), that is, as the beam‐on time increases, results approach those for non‐gated delivery.

### 3.2 Beam profile

**FIGURE 4:**
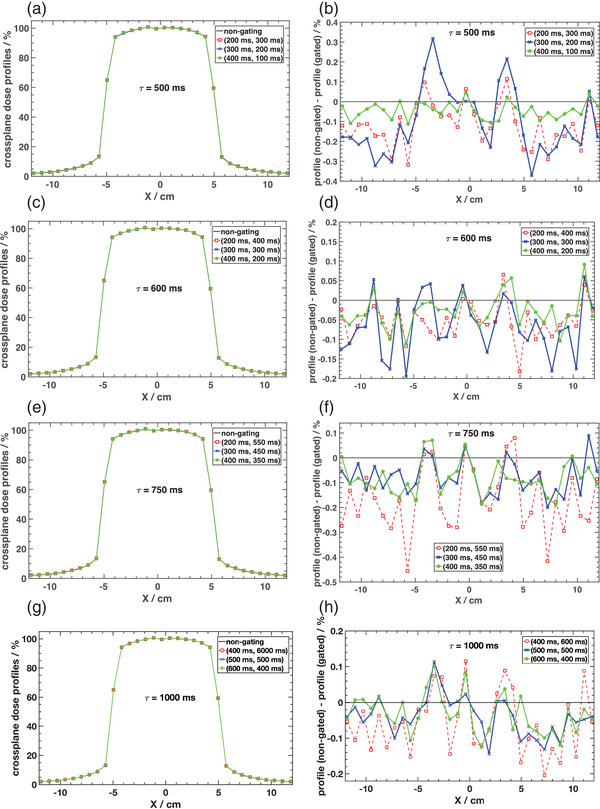
Crossplane beam profiles normalized to central axis dose and differences relative to non‐gated delivery for a 10 × 10 cm^2^ field measured with the IBA MatriXX detector array into the MultiCube phantom and with SAD = 100 cm. Plots are shown for different (beam‐on, beam‐off) times and for cardiac cycle periods of 500 ms (a and b), 600 ms (c and d), 750 ms (e and f), and 1000 ms (g and h).

Figure 4 shows the comparison of beam profiles measured with the IBA MatriXX detector array in the MultiCube phantom between non‐gated beam delivery and various gating windows. Figure 4a gives measurements for a cardiac cycle of 500 ms with (beam‐on, beam‐off) times varying from (200, 300 ms) to (400, 100 ms). Differences relative to the non‐gated mode are given in Figure 4b and are within 0.4% for all gating windows investigated even outside of the geometric bounds of the field.

For a cardiac cycle of 600 ms, maximum differences were <0.2% relative to the non‐gated delivery mode as shown in Figure 4c,d. Results for a heart rate interval of 750 ms are shown in Figure 4e,f with a maximum difference of ∼0.5% when using a beam‐on time of 200 ms. Similar results were also found when using a cardiac cycle of 1000 ms and different gating windows with differences to non‐gated mode within 0.2% as shown in Figure 4g,h.

**FIGURE 5:**
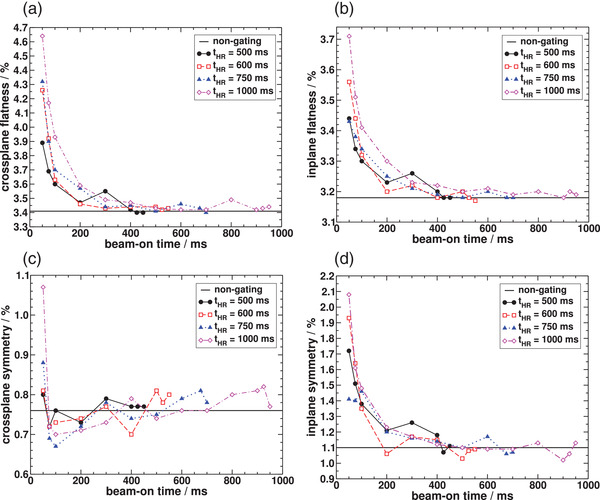
Flatness (a and b) and symmetry (c and d) calculated for the beam profiles measured with the IBA MatriXX detector array into the MultiCube phantom using a 10 × 10 cm^2^ field and with SAD = 100 cm. Values are shown as a function of beam‐on time for cardiac cycles of 500, 600, 750, and 1000 ms in comparison to the non‐gated (base line) beam delivery.

Analysis of the beam profiles can also be performed by quantifying the flatness and symmetry for the different gating windows used, with comparison to non‐gated beam delivery. Figure 5a,b shows the variation of flatness with beam‐on time in the crossplane an in the inplane dimensions, respectively. Differences in flatness relative to the non‐gated delivery decrease as the beam‐on time increases relative to beam‐off time. For all cardiac cycles investigated, most differences are <5% for beam‐on time ≥200 ms. A similar behavior is observed for crossplane and inplane symmetry as shown in Figure 5c,d, respectively. The symmetries of the profiles for the gated beams were found to converge to the value obtained for non‐gated delivery as the beam‐on time was increased above 200 ms.

### 3.3 Real‐time cardiac gating

**FIGURE 6:**
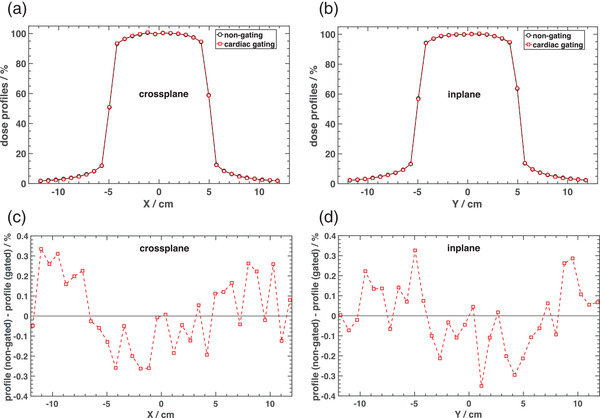
Crossplane (a) and inplane (b) dose profiles obtained in non‐gated mode beam delivery and gating the beam to real‐time electrocardiogram (ECG) signal. Dose profile differences are also shown for both crossplane (c) and inplane (d) directions.

Figure 6 shows a comparison of dose profiles (a and b) measured for non‐gated delivery and with real‐time ECG‐synchronized gating delivery for a 10 × 10 cm^2^ single field normally incident on the IBA MatriXX and the MultiCube phantom. Differences between the dose profiles for the two acquisition modes were <0.4% in both *x* (crossplane) and *y* (inplane) directions as shown in Figure 6c,d.

Although the beam‐on time is constantly changing in response to variations in heart rate time, actual beam‐on times were within the range of gating windows investigated in this work. Therefore, no significant differences would be expected when gating the beam based on an actual ECG signal, as compared to the non‐gated delivery as we have demonstrated in previous sections.

**FIGURE 7:**
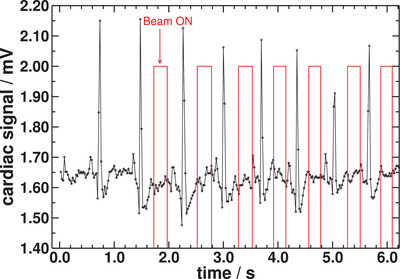
Electrocardiogram (ECG) signal recorded during the first 6 s while delivering the single field gated plan presented in Figure 6. R–R intervals and beam‐on times are constantly updated based of information of current and previous R‐peaks. Therefore, synchronization is maintained by turning the beam on *τ*/3 ms after the last R‐peak. The graph shows variation in cardiac cycle period with minimum and maximum heart rates of 77 and 95 bpm, respectively

Figure 7 shows the ECG signal recorded while delivering the single field plan gated in synchronization with heart rate variation. The graph also shows the instants at which the beam is turned on immediately after each R‐peak based on the respective calculated cardiac cycle period. Regardless of heart rate variation, irradiation is maintained within the quiescent interval of the heart.

**FIGURE 8:**
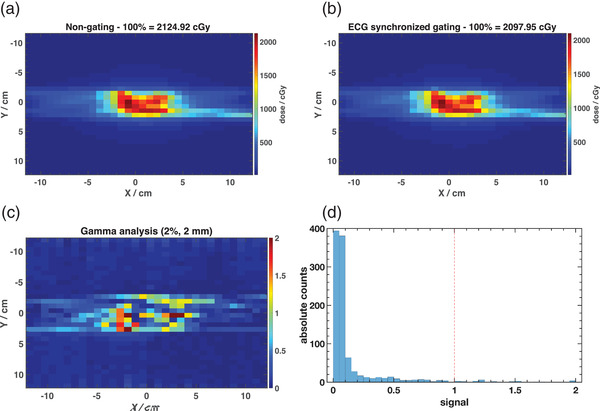
Comparison of intensity‐modulated radiation therapy (IMRT) dose distribution for non‐gated (a) and real‐time electrocardiogram (ECG)‐synchronized gating delivery (b) measured with the IBA MatriXX and the MultiCube phantom. A gamma analysis result with 2% and 2 mm criteria is shown in (c) with the corresponding histogram of gamma values (d).

Figure 8 shows IMRT dose distributions measured with the IBA MatriXX and the MultiCube phantom for the non‐gated mode (a) and real‐time ECG‐synchronized gated delivery (b). A gamma analysis performed on non‐normalized dose distributions, that is, dose values in Gy, with 2% dose and 2 mm spatial criteria was used to compare the dose distributions, with results shown in Figure 8c,d. This yielded a passing rate of 97.2%, demonstrating a good agreement between the ECG‐synchronized gating treatment delivery and that obtained in the non‐gated mode.

When using a more stringent criteria of 1% and 1 mm, the passing rate decreases to 92.3%. In practice a passing rate ≥90% would be considered acceptable with a 3% and 3 mm criteria,^34^ which therefore confirms the good agreement between the dose distributions.

## 4 DISCUSSION

Results obtained for dose linearity show that the LINAC beam output remains linear for most gating windows investigated and for the range of cardiac cycle period between 500 ms (120 beats/min) and 1000 ms (60 beats/min), which corresponds to typical human heart rate. Figure 3 shows that dose linearity is worse in the range of small MU settings (<100 MU) regardless of the gating mode, although they are still within 2%. We observe, for example, from Figure 3a, that even for the non‐gated mode, there is a variation of 0.5% in the chamber response when the amount of MUs delivered varies from 10 to 40 MUs. In comparison, a variation of only 0.1% is observed in the chamber response, within the same MU range, when gating the beam within 500 ms period with a d.c. of 40% (beam on = 200 ms). Although a 2% variation in the chamber response was observed for the largest period of 1000 ms with 40% d.c. between 10 and 40 MUs, it is not possible to infer that linearity is always degraded with shorter cardiac periods. Comparison between Figure 3b,c in the same MU range, for example, shows an improvement in the chamber response variation from 0.6% for the 600 ms period with 33% d.c to 0.1% for the 750 ms period with 27% d.c. For completeness, dose linearity should also be evaluated below 10 MUs, as for IMRT or VMAT dose modulation, sometimes, smaller MUs may be delivered. However, it would have a negligible impact on single fraction SBRT treatments for VT that usually presents minimum values of MUs per segment >10 MUs.

Similar to what was observed for dose linearity, the agreement among dose profiles improves as the d.c. approaches the non‐gated scenario. Symmetry and flatness of beam profiles are not strongly affected for most gating windows investigated, and particularly when using beam‐on times ≥200 ms. Deviations in flatness and symmetry from the non‐gated mode when using beam‐on times much <200 ms suggest a limitation when gating the beam in that range, possibly due to the lack of enough profile data acquired in such short time. However, a more detailed investigation on that, using different LINACs, would be necessary to thoroughly address this issue. As beam‐on times would typically be >200 ms, such investigation was not elected as a priority in this work and constitutes one of the limitations of this study.

Results obtained with a real‐time ECG signal acquisition to gate the beam synchronized with heart rate variations as shown in Figures 6 and 8 confirm that the proposed methodology does not provide significant differences in beam stability and dose distribution as compared to normal mode delivery. However, despite using an IMRT with maximum dose rate of 600 MU/min, when gating the beam with the ECG signal, maximum dose rate achieved is ∼300 MU/min. Therefore, the dose rate change between segments has not caused any issues other than increasing the total delivery time of the gated plan as compared to the normal one. Total delivery time for the non‐gated and ECG gated modes was ∼18 and 34 min, respectively. This limitation can be overcome in a future work when implementing this technique in a TrueBeam machine and using VMAT as a delivery technique.

These results confirm that using an IMRT plan with real‐time cardiac synchronized gating has minimal impact on the dose delivered and can therefore be explored further to improve the outcomes in SBRT of VT.

Main limitations of this study include a lack of correlation between target position and a surrogate such as the ECG signal to accurately assess dose delivery, as well as accounting for respiratory motion combined with intrinsic heart movement and temporal accuracy of the gating signal relative to beam on/off (latency assessment). An implication of this is that the amplitude of the motion of the target as a function of the heart rate was not addressed in this current study, as we wanted to evaluate the impact of typical cardiac gating windows in a static dose distribution. Evaluation of energy constancy under such high frequencies is also recommended, and it will be accounted for in a future investigation. Use of a phantom with realistic cardiac and respiratory motion represents a crucial step for validating a gated VT‐SBRT technique to overcome the main limitations of this study and constitutes our main motivation in future work. Recent publications in the literature^18^, ^35^, ^36^ can provide useful insight to account for combined cardiac and respiratory motions. Additionally, as most cardiac VT treatments are performed on TrueBeam platforms and using VMAT techniques, this will be examined in future work.

## 5 CONCLUSIONS

In this work, with the anticipated application to SBRT for VT, we investigated the feasibility of gating a radiation beam synchronized to the cardiac cycle by using a real‐time ECG signal. Our results demonstrate that dose linearity, beam flatness, and symmetry are not strongly affected when gating the beam at high frequencies within typical human cardiac cycles. We provide data showing the trend of deviation of beam flatness and symmetry as gated beam‐on times are reduced below low values, for example, <200 ms. We demonstrate a robust algorithm for detecting the heart rhythm from ECG that is capable of adjusting the timing of gating with variable heart rate. Comparison of dose distributions between non‐gated and cardiac‐gated delivery show acceptable agreement for both static beam and IMRT plans.

## CONFLICT OF INTEREST

Dr. Reis has nothing to disclosure. Dr. Robar reports grants from Varian Medical Systems, other from Adaptiiv Medical Technologies, outside the submitted work.

## AUTHOR CONTRIBUTION

Cristiano Q. M. Reis: measurements, software development (ECG controlled beam gating), contouring, treatment planning, data analysis, and manuscript preparation.

James L. Robar: study conception, analysis, manuscript review, and project supervision.

All authors discussed the results and contributed to the manuscript.
